# Trimethylamine-*N*-Oxide (TMAO)-Induced Impairment of Cardiomyocyte Function and the Protective Role of Urolithin B-Glucuronide

**DOI:** 10.3390/molecules23030549

**Published:** 2018-03-01

**Authors:** Monia Savi, Leonardo Bocchi, Letizia Bresciani, Angela Falco, Federico Quaini, Pedro Mena, Furio Brighenti, Alan Crozier, Donatella Stilli, Daniele Del Rio

**Affiliations:** 1 Department of Chemistry, Life Sciences and Environmental Sustainability, University of Parma, Parco Area delle Scienze 11/A, 43124 Parma, Italy; monia.savi@unipr.it (M.S.); leonardo.bocchi@unipr.it (L.Bo.); 2 Department of Veterinary Science, University of Parma, Strada del Taglio 10, 43126 Parma, Italy; letizia.bresciani@unipr.it; 3 Department of Medicine and Surgery, University of Parma, Via A. Gramsci 14, 43126 Parma, Italy; angela.falco@unipr.it (A.F.); federico.quaini@unipr.it (F.Q.); 4 Department of Food and Drugs, University of Parma, Parco Area delle Scienze 27/A, 43124 Parma, Italy; pedromiguel.menaparreno@unipr.it (P.M.); furio.brighenti@unipr.it (F.B.); 5 Department of Nutrition, University of California, 3143 Meyer Hall One Shields Avenue, Davis, CA 95616-5270, USA; alan.crozier44@gmail.com

**Keywords:** ellagitannins, cardiomyocyte mechanics, cardiovascular diseases, Trimethylamine-*N*-oxide, urolithins

## Abstract

One of the most recently proposed candidates as a potential trigger for cardiovascular diseases is trimethylamine-*N*-oxide (TMAO). Possible direct effects of TMAO on myocardial tissue, independent of vascular damage, have been only partially explored so far. In the present study, we assessed the detrimental direct effects of TMAO on cardiomyocyte contractility and intracellular calcium dynamics, and the ability of urolithin B-glucuronide (Uro B-gluc) in counteracting TMAO-induced cell damage. Cell mechanics and calcium transients were measured, and ultrastructural analysis was performed in ventricular cardiomyocytes isolated from the heart of normal adult rats. Cells were either untreated, exposed to TMAO, or to TMAO and Uro B-gluc. TMAO exposure worsened cardiomyocyte mechanics and intracellular calcium handling, as documented by the decrease in the fraction of shortening (FS) and the maximal rate of shortening and re-lengthening, associated with reduced efficiency in the intracellular calcium removal. Ultrastructurally, TMAO-treated cardiomyocytes also exhibited glycogen accumulation, a higher number of mitochondria and lipofuscin-like pigment deposition, suggesting an altered cellular energetic metabolism and a higher rate of protein oxidative damage, respectively. Uro B-gluc led to a complete recovery of cellular contractility and calcium dynamics, and morphologically to a reduced glycogen accumulation. We demonstrated for the first time a direct negative role of TMAO on cardiomyocyte functional properties and the ability of Uro B-gluc in counteracting these detrimental effects.

## 1. Introduction

Non-communicable diseases (NCDs) are the leading cause of death and kill 38 million people each year. Cardiovascular diseases (CVDs) account for most of the NCD deaths, i.e., 17.5 million people annually, followed by cancers (8.2 million), respiratory diseases (4 million), and diabetes (1.5 million) [1].

Recent data reinforce the importance of dietary habits in maintaining cardiovascular health [2]. Metabolomics, which constitutes an exciting new research area, aims at identifying small-molecule metabolites that may act as potential triggers for CVDs. The most recently proposed candidate in this framework is trimethylamine-*N*-oxide (TMAO), a compound produced by liver through a flavin-monooxygenase 3 (FMO3) oxidation of the gut microbiota-derived trimethylamine (TMA). TMA appears to originate from two main sources, namely phosphatidylcholine/choline and carnitine [3]. Although the available studies on the role of TMAO in CVD development are limited and discordant [4,5], it has been suggested that the intake of these dietary compounds, often found in large quantities in animal products as red meat, fish and eggs, may be a critical factor in promoting cardiovascular risk, mainly related to the inception and progression of atherosclerosis, in both rodents and humans [6,7,8,9]. Nevertheless, potential direct effects of TMAO on myocardial tissue, independent of vascular damage, have been only partially explored so far [10,11].

As recently reviewed by Velasquez and colleagues [12], efforts have been directed to verifying the effects of some main strategies for preventing TMAO damages to the human body, namely: (i) limiting the intake of phosphatidylcholine/choline and/or carnitine rich food [6]; (ii) targeting the microbiota composition to limit TMA production through functional ingredients such as probiotics [13,14], through specific natural occurring compounds [15,16], through targeted pharmacologic interventions [6,9], through antibiotics [7], or through colonization of human proximal gut with selected bacteria [17,18]; and (iii) inhibiting FMO3 as the main enzyme involved in TMA oxidation [19].

An additional viable strategy that should be explored to prevent TMAO-related pathologies is identifying compounds that circulate in vivo after the intake of specific foods and are able to counteract the actions of this molecule towards the cardiovascular system. Dietary polyphenols could constitute valid candidates for this purpose. They are ubiquitous in plant foods and beverages and have been widely explored for their many biological activities [20,21]. The contribution of polyphenols to cardioprotection has been largely described, but is still not completely understood [22,23,24,25]. A polyphenolic subclass, ellagitannins (ETs), found in some fruits and nuts, are attracting increasing interest due to their many reported positive effects toward human health [26]. Urolithins, which are the main colonic metabolites generated by microbial metabolism of ETs [27], persist at relatively high concentrations in plasma and urine for days after ingestion of dietary ETs, and exhibit anti-inflammatory and anti-atherosclerotic activities, supporting the hypothesis of a potential preventive effect against CVDs [26,28]. We previously demonstrated that different urolithins are able to reduce the pro-inflammatory mediators secreted by cultured neonatal rat cardiomyocytes and fibroblasts exposed to high glucose concentrations, suggesting that the cellular inflammatory response to hyperglycemia could be attenuated by the regular intake of ET-rich foodstuffs [29]. Specifically, we found that urolithin B (Uro B) and urolithin B-glucuronide (Uro B-gluc) succeeded in preventing inflammatory responses in cardiomyocytes. Moreover, in a recent in vivo study, we showed that intraperitoneal urolithin administration results in a reduced inflammation in myocardial tissue and a significant recovery of cardiac function in a rat model of early diabetes (3 weeks of hyperglycemia) [25].

The present study was specifically designed to: (i) demonstrate, for the first time, the capacity of TMAO to directly impair cardiomyocyte contractile function and intracellular calcium dynamics, and (ii) verify the ability of Uro B-gluc exposure to reduce TMAO-induced cell damage. Uro B-gluc, one of the main metabolites in circulation after ET consumption, was chosen because of its positive effects on inflammation and cardiomyocyte morphofunctional properties [25,29], and its weak intracellular deglucuronidation previously demonstrated in cardiomyocytes and primary human aortic endothelial cells [29,30].

## 2. Results

### 2.1. TMAO-Induced Detrimental Effects on Cardiomyocyte Functional Properties Were Reverted by the Simultaneous Exposure to Uro B-Gluc

**Figure 1:**
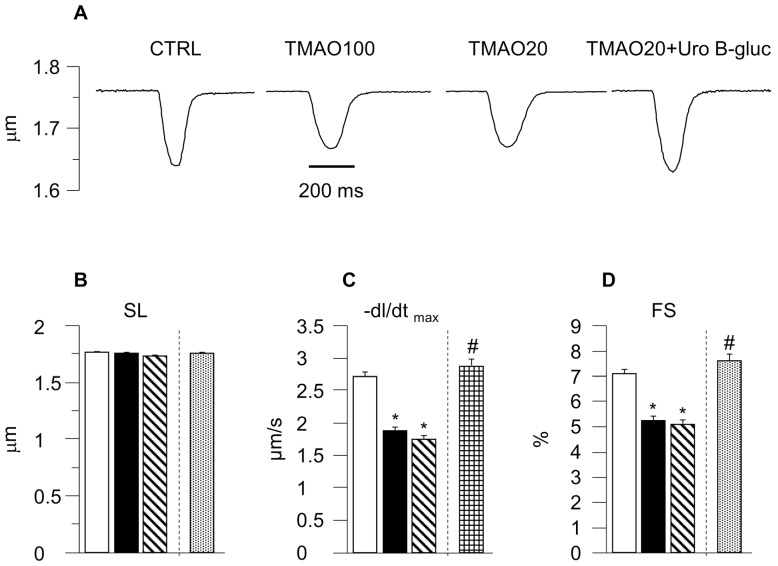

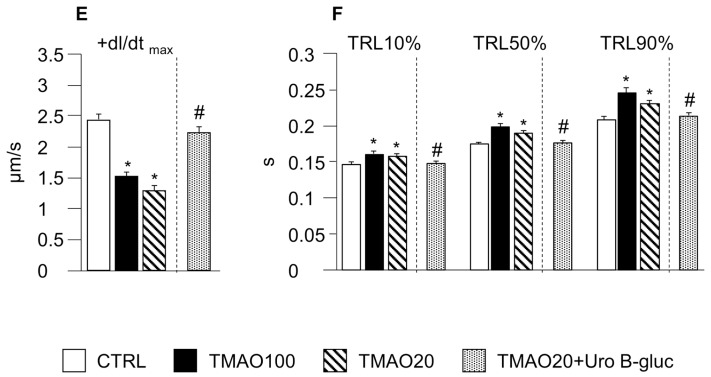
Cell mechanics. Representative examples of sarcomere shortening (SL) (**A**) recorded from CTRL, TMAO100, TMAO20 and TMAO20 + Uro B-gluc ventricular myocytes. In bar graphs (**B**–**F**): mean values ± SEM of diastolic sarcomere length (SL; **B**), maximal rate of shortening (−dl/dt_max_; **C**), fraction of shortening (FS; **D**), maximal rate of re-lengthening (+dl/dt_max_; **E**), time at 10%, 50%, and 90% of re-lengthening (TRL10%, TRL50%, TRL90%; **F**), measured in CTRL (n = 115), TMAO100 (*n* = 84), TMAO20 (*n* = 100), and TMAO20 + Uro B-gluc (*n* = 82) cells. * *p* < 0.05 significant differences vs. CTRL; # *p* < 0.05 significant differences vs. TMAO20. General Linear Model/ANOVA GLM/ANOVA for repeated measurements was used first to compare CTRL, TMAO100, and TMAO20 groups to evaluate TMAO dose-dependent effects, and then CTRL, TMAO20, and TMAO20 + Uro B-gluc groups, after selecting the TMAO minimal effective dose.

**Figure 2:**
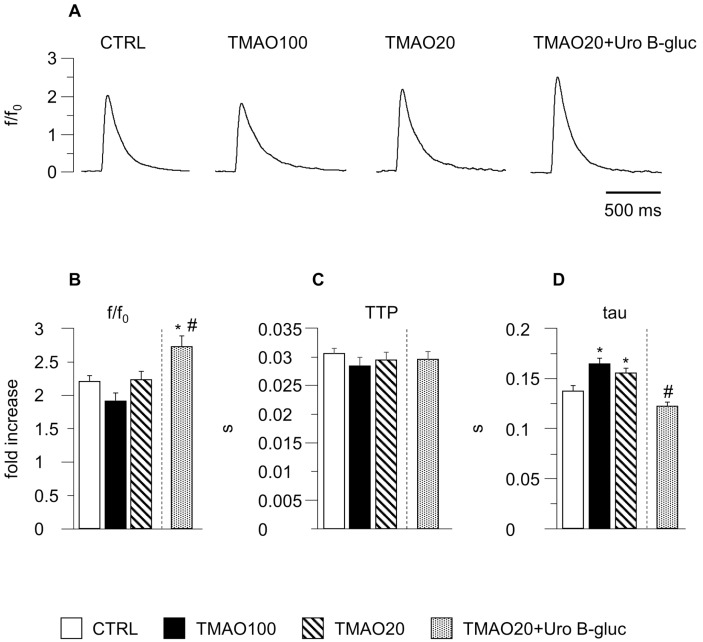
Intracellular calcium transients. Representative examples of calcium transients (**A**), normalized traces: fold increase, f/f0) recorded from CTRL, TMAO100, TMAO20 and TMAO20 + Uro B-gluc ventricular myocytes. In bar graphs (**B**–**D**): mean values ± SEM of Ca^2+^ transient amplitude expressed as peak fluorescence normalized to baseline fluorescence (f/f0; **B**), time to peak of the calcium transient (TTP; **C**), and time constant of the intracellular calcium decay (tau; **D**), measured in CTRL (*n* = 50), TMAO100 (*n* = 33), TMAO20 (*n* = 35), and TMAO20 + Uro B-gluc (*n* = 29) cardiomyocytes. * *p* < 0.05 significant differences vs. CTRL; # *p* < 0.05 significant differences vs. TMAO20 (GLM/ANOVA, other explanations as in Figure 1).

The results obtained in the present study suggested that (1) TMAO negatively affected cardiomyocyte mechanics and calcium dynamics, independently of the dose; and (2) adding Uro B-gluc to the lowest effective dose of TMAO (20 µM) completely restored cardiomyocyte functional properties (Figure 1 and Figure 2). Specifically, the average diastolic sarcomere length (SL) was similar in control cells (CTRL), in cells treated with TMAO at 100 μM (TMAO100) and in cells treated with TMAO at 20 μM (TMAO20) (Figure 1B), whereas contraction/relaxation properties and intracellular calcium dynamics measured in unloaded ventricular myocytes treated with TMAO, at both concentrations, were considerably impaired compared to CTRL (Figure 1C–F). TMAO100 and TMAO20 significantly decreased the sarcomere fraction of shortening (FS, −26% and −28% respectively; Figure 1D), as well as the maximal rate of shortening (−dl/dt_max_, −31% and −35%; Figure 1C) and re-lengthening (+dl/dt_max_, −38% and −47%; Figure 1E), leading to a significant prolongation of re-lengthening times (time at 10%, 50% and 90% of re-lengthening: TRL10%, TRL50%, and TRL90%; Figure 1F).

The impaired cell contraction in TMAO100 and TMAO20 exposed cells was associated with a prolongation in the time required to remove cytosolic Ca^2+^ (tau, +20% and +13% respectively; Figure 2D) while the amplitude of the calcium transient (peak fluorescence normalized to baseline fluorescence: f/f0) and the time to peak (TTP) were comparable in the three experimental groups, with only minor changes (Figure 2B,C).

Uro B-gluc led to a complete recovery of cellular contractility and calcium dynamics (Figure 1A,C–F and Figure 2A–D). Noteworthy, the amplitude of the calcium transient in TMAO20 + Uro B-gluc exposed cells was significantly higher than CTRL and TMAO20 groups by +24% and +22% respectively (Figure 2B). Consistent with this finding, the values of the FS increased after Uro B-gluc exposure by 7% and 49%, in comparison with CTRL and TMAO20, respectively (Figure 1D).

### 2.2. Transmission Electron Microscopy (TEM) Analysis

**Figure 3:**
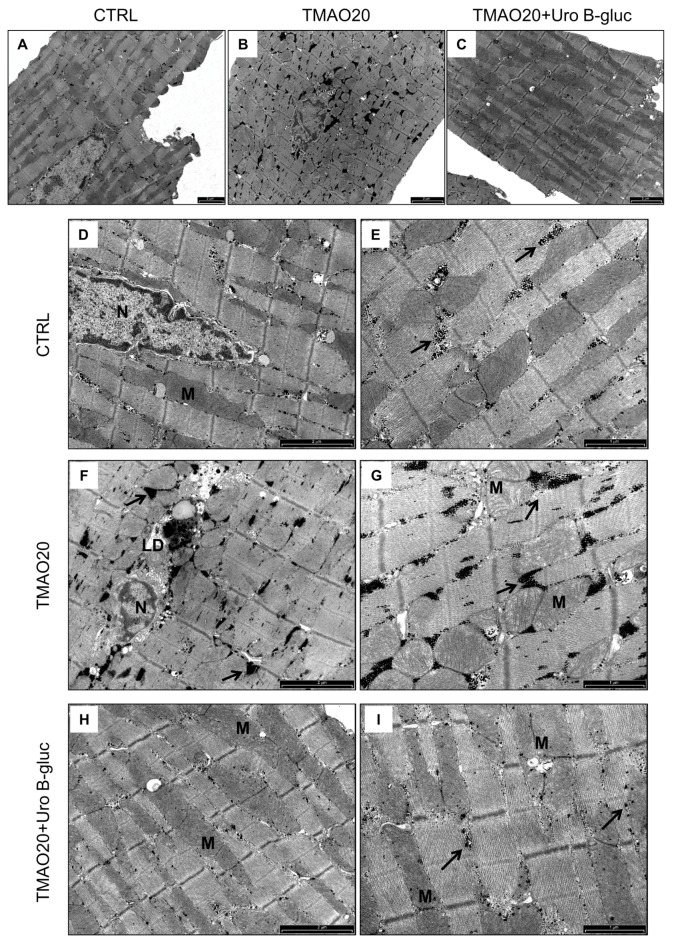
Effects of TMAO and TMAO + Uro B-gluc on cardiomyocyte ultrastructure (TEM study). (**A**–**C**): low magnification TEM images of isolated cardiomyocytes in the absence (**A**) or presence of TMAO alone (**B**) or in combination with Uro B-gluc (**C**). (**D**,**E**): CTRL untreated cardiomyocytes showing a regular distribution of mitochondria and glycogen (arrows) between sarcomeric units; (**F**,**G**): the in vitro exposure of cardiomyocytes to TMAO induced accumulation of glycogen (arrows), occasional lipofuscin-like deposition (LD) and mitochondria enlargement. (**H**,**I**): the addition of Uro B-gluc to TMAO reduced glycogen compartmentalization without changes in mitochondrial size compared to TMAO alone. M: Mitochondria; N: cardiomyocytenucleus. Scale bars: 2 µm (**A**–**D**,**F**,**H**); 1 µm (**E**,**G**,**I**).

**Figure 4:**
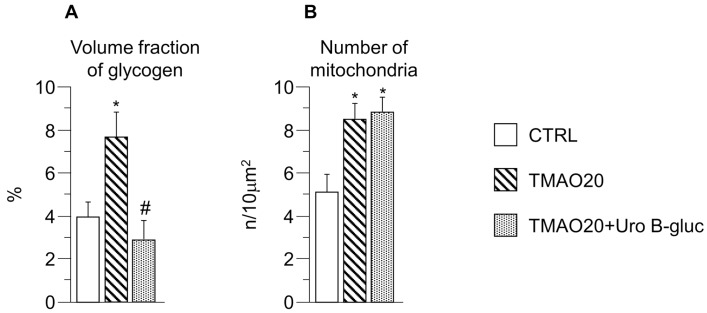
Effects of TMAO and TMAO + Uro B-gluc on cardiomyocyte glycogen content and mitochondria. Mean values ± SEM of the volume fraction of glycogen (**A**, %) and the number of mitochondria (**B**, n/10 μm^2^) in CTRL, TMAO20, and TMAO20 + Uro B-gluc cells. * *p* < 0.05 significant differences vs. CTRL; # *p* < 0.05 significant differences vs. TMAO20 (non-parametric statistical test: Kruskal Wallis and U-Mann Whitney test).

TEM images of cardiomyocytes in CTRL, TMAO20 and TMAO20 + Uro B-gluc group, taken at low and high magnification, are reported in Figure 3A–C (low magnification) and D–I (high magnification). Ultrastructural analysis of isolated cardiomyocytes revealed that cells exposed to TMAO20 exhibited compartmentalization and significant increase in glycogen accumulation with respect to CTRL cells (+94%; Figure 3D–G and Figure 4A). This finding was associated with a higher number of mitochondria (approximately +67%; Figure 3A,B and Figure 4B), in the absence of substantial changes in the volume fraction of both myofibrils and mitochondria. In addition, paranuclear deposition of lipofuscin-like pigment was detected following TMAO exposure (Figure 3F). The simultaneous treatment with TMAO 20 µM and Uro B-gluc (TMAO20 + Uro B-gluc) induced a significant reduction in glycogen accumulation (Figure 3H,I and Figure 4A) without affecting mitochondrial number (Figure 3C and Figure 4B).

## 3. Discussion

High circulating concentration of TMAO has recently been associated with different cardiac and vascular pathologies [31] and, in this framework, several studies have tried to elucidate the contribution of TMAO to the development of CVDs [10,11,32,33,34,35]. However, the mechanisms behind its pathogenic effects remain unclear and largely speculative. Furthermore, population-wide studies are limited and extremely discordant [4,5].

To the best of our knowledge, no attempts have been made to investigate the potential acute and direct detrimental effects of TMAO on cardiomyocytes and the ability of diet-derived circulating compounds to counteract its negative actions. Our results demonstrated, for the first time, that (i) TMAO is able to negatively affect cardiomyocyte contractility and intracellular calcium dynamics, and (ii) the co-treatment with Uro B-gluc is able to induce a complete recovery of cardiomyocyte functional properties.

Although other mechanisms cannot be ruled out, alterations in cardiomyocyte mechanics can be attributed to the TMAO-induced early cellular pro-inflammatory response and mitochondrial damage leading to a reduced energy production, as documented in previous studies [10,11]. Indeed, it has been shown that cardiomyocytes exposed to environmental challenges over-express pro-inflammatory cytokines [11,23], which can directly affect the intracellular contractile machinery by activating specific molecular pathways [11,36,37]. In addition, glycogen accumulation in cardiomyocytes, as detected by transmission electron microscopy (TEM), could be due to TMAO-induced inhibition of pyruvate dehydrogenase (PDH), resulting in a reduced pyruvate oxidation in cardiac mitochondria and lower ATP production [10]. This defective glycolysis might, in turn, divert glucose utilization to other routes, including glycogen synthesis [38]. Lipofuscin-like pigments in the paranuclear area of cardiomyocytes were also observed in TMAO-treated cells. Lipofuscin formation is known to be linked to the rate of protein oxidative damage, and the functional impairment of mitochondria, as well as proteasomal system and lysosomes [39]. Lipofuscin is not an inert material but may have a chemically very reactive surface that can disturb cellular metabolism [39]. The presence of lipofuscin is also used as an indicator of oxidative damage in biological systems [40]. A TMAO-induced mitochondrial dysfunction following cell oxidative and inflammatory damage can lead to altered energy production and is consistent with our results, showing that contractile parameters, strictly depending on ATP availability, were worsened in TMAO-treated cardiomyocytes.

The “inflammation hypothesis” is somehow supported by the results obtained in cardiomyocytes co-treated with Uro B-gluc, as this molecule has proven anti-inflammatory effects [25,29] and might have been able to counteract the TMAO pro-inflammatory action, resulting in a recovery of cell contractility and intracellular calcium dynamics, as we actually observed. On the other hand, one can speculate that Uro B-gluc, as with other polyphenols like resveratrol, could activate SIRT1, which reduces the activity of Forkhead box protein O1 (Foxo1), leading to increased glycolysis through a positive modulation of PDH activity [41], justifying the reduced glycogen accumulation in TMAO20 + Uro B-gluc cardiomyocytes. In addition, protein sirtuin 1 (SIRT1) activation is known to increase the sarcoplasmic reticulum (SR) calcium ATPase 2 (SERCA2) expression, improving cardiac function in different heart diseases, such as diabetic cardiomyopathy [42].

A higher Uro B-gluc-induced expression/activity of SERCA2 leading to an increased SR calcium content results in an increased amplitude of the Ca^2+^ transient, as we actually observed in the co-treated cardiomyocytes compared to both TMAO and CTRL cells. The enhanced amount of calcium released was associated with a complete recovery of the rate of calcium clearing (tau values), thus preventing alterations in the intracellular calcium homeostasis that can lead to both contractile and electrophysiological consequences [43,44]. Indeed, the rate of Ca^2+^ transient recovery is commonly measured as a mono-exponential decay which assumes that Ca^2+^ removal, at any time, depends on the cytoplasmic Ca^2+^ concentration, and the time constant of its exponential decay, in the absence of changes in removal mechanisms, is constant, independently of the amplitude of the transient [45].

## 4. Materials and Methods

### 4.1. Experimental Animals

Experiments were performed on cardiomyocytes isolated from 12 male Wistar rats bred in our departmental animal facility, aged 12–14 weeks and weighing 350–400 g. The animals were kept in single-sex groups of four individuals from weaning (4 weeks after birth) until the onset of the experiments, in a temperature-controlled room at 20–24 °C, with the light on between 7.00 a.m. and 7.00 p.m. The bedding of the cages consisted of wood shavings; food and water were freely available. The investigation was approved by the Veterinary Animal Care and Use Committee of the University of Parma-Italy (Prot. No. 59/12) and conforms to the National Ethical Guidelines of the Italian Ministry of Health and the Guide for the Care and Use of Laboratory Animals (National Institute of Health, Bethesda, MD, USA, revised 1996). All experiments were carried out in accordance with the approved guidelines.

### 4.2. Isolation of Adult Left Ventricular Cardiomyocytes

Individual ventricular myocytes were enzymatically isolated by collagenase perfusion in accordance with a procedure previously described [46]. Briefly, each rat was anesthetized with a mixture of ketamine chloride (40 mg/kg i.p.; Imalgene, Merial, Italy) and medetomidine hydrochloride (0.15 mg/kg ip; Domitor, Pfizer Italia s.r.l., Latina, Italy). Then, the heart was rapidly excised through a median sternotomy, mounted on a Langendorff apparatus and perfused at 37 °C with a sequence of solutions gassed with 100% O_2_: (i) calcium-free solution for 5 min to remove the blood; (ii) low-calcium solution (0.1 mM) plus 1 mg/mL type 2 collagenase (Worthington Biochemical, Lakewood, NJ, USA), and 0.1 mg/mL type XIV protease (Sigma-Aldrich, Milan, Italy) for about 20 min; and (iii) enzyme-free low-calcium solution for 5 min. Calcium-free solution contained the following (in mM): 126 NaCl, 22 dextrose, 5.0 MgCl_2_, 4.4 KCl, 20 taurine, 5 creatine, 5 Na pyruvate, 1 NaH_2_PO_4_, and 24 HEPES (pH = 7.4, adjusted with NaOH) (all chemicals from Sigma-Aldrich). The left ventricle was then minced, gently agitated for 5 min, and filtered through a nylon mesh. Eventually, cells were re-suspended in low-calcium solutions (0.1 mM) for 1 h at room temperature and used for measuring sarcomere shortening and calcium transients. At the end of the experiments, the remaining cardiomyocytes were washed twice in low-calcium solution (0.1 mM), fixed in Karnovsky solution (4% formaldehyde, 5% glutaraldehyde) for 3 h at room temperature, and used for TEM study.

### 4.3. TMAO and Uro B-Gluc Treatments

TMAO (Alfa Aesar, Thermo Fisher (Kandel) GmbH, Karlsruhe, Germany) was dissolved in low-calcium (0.1 mM) maintenance solution to obtain a 10 mM stock solution.

In the first part of the study, freshly isolated cardiomyocytes from 5 rats were incubated for two hours with buffered maintenance solution without TMAO, or with TMAO at two different concentrations, 100 μM (TMAO100) and 20 μM (TMAO20), in order to discover dose-dependent effects of the compound on cardiomyocyte function. Both doses were comparable to the TMAO plasmatic concentrations reached after a l-carnitine challenge in mice and in omnivore subjects, respectively [6].

In the second part of the study, isolated cardiomyocytes from the 7 remaining rats were incubated for two hours with buffered maintenance solution with TMAO 20 μM, the lower effective dose discovered in the first part of the study, or TMAO 20 μM plus Uro B-gluc 10 μM (TMAO20 + Uro B-gluc). Uro B-gluc was provided by Olivier Dangles (INRA, Avignon, France), and synthesized according to Tognolini et al. [47]. The 10 µM concentration is comparable to the circulating concentration obtained after consumption of pomegranate juice [48], and similar to those previously adopted by our group for in vitro studies investigating urolithin bioactivity [49].

### 4.4. Cardiomyocyte Contractility and Ca^2+^ Transients

Mechanical properties of treated and untreated left ventricular myocytes were assessed by using the IonOptix fluorescence and contractility system (IonOptix, Milton, MA, USA). Cardiomyocytes were placed in a chamber mounted on the stage of an inverted microscope (Nikon-Eclipse TE2000-U, Nikon Instruments, 40X oil objective, NA: 1.3; Florence, Italy) and superfused (1 mL/min at 37 °C) with a Tyrode solution containing (in mM): 140 NaCl, 5.4 KCl, 1 MgCl_2_, 5 HEPES, 5.5 glucose, and 1 CaCl_2_ (pH 7.4, adjusted with NaOH) (all chemicals from Sigma-Aldrich). Only rod-shaped myocytes with clear edges and average sarcomere length ≥1.7 µm were selected for the analysis. All the selected myocytes did not show spontaneous contractions. The cells were field-stimulated at a frequency of 0.5 Hz by constant current pulses (2 ms in duration, and twice diastolic threshold in intensity) delivered by platinum electrodes placed on opposite sides of the chamber, connected to a MyoPacer Field Stimulator (IonOptix). The stimulated myocyte was displayed on a computer monitor using an IonOptix MyoCam camera. Load-free contraction of myocytes was measured with the IonOptix system, which captures sarcomere length dynamics via a Fast Fourier Transform algorithm.

A total of 381 isolated ventricular myocytes were analyzed (115 control cells (CTRL), 84 TMAO100, 100 TMAO20, and 82 TMAO20 + Uro B-gluc) to assess cellular mechanical properties. The following parameters were computed: mean diastolic SL, FS, maximal rates of shortening and re-lengthening (±dl/dt_max_), and time at 10%, 50%, and 90% of re-lengthening (TRL10%, TRL50%, and TRL90%). Steady-state contraction of myocytes was achieved before data recording. Sampling rate was set at 1 KHz.

In a subset of cells of each group (50 CTRL, 33 TMAO100, 35 TMAO20, and 29 TMAO20 + Uro B-gluc), Ca^2+^ transients were measured simultaneously with cell motion. Ca^2+^ transients were detected by epifluorescence after loading the myocytes with fluo-3-AM (10 µM; Invitrogen, Carlsbad, CA, USA) for 30 min. Excitation wavelength was 480 nm, with emission collected at 535 nm. Fluo-3 signals were expressed as normalized fluorescence (f/f0: fold increase). The time to peak of the calcium transients (TTP) was measured, as indirect index of ryanodine receptor efficiency. The time course of the fluorescence signal decay was described by a single exponential equation, and the time constant (tau) was used as a measure of the rate of intracellular Ca^2+^ clearing [45].

### 4.5. TEM Study

Fixed cardiomyocytes were washed several times with 0.1 M phosphate buffer (pH 7.2), post-fixed in 1% osmium tetroxide (OsO_4_) for 90 min at room temperature, and dehydrated by increasing concentration of alcohol. Then, cells were washed with propylene oxide and embedded in epoxy resin. Sections of 0.5 μm thickness were stained with methylene blue and safranin to morphologically select the field of interest. Subsequently, ultrathin 60–80 nm thick sections were collected on a 300-mesh copper grid and, after staining with uranyl acetate and lead citrate, were qualitatively examined under a transmission electron microscope (Philips EM 208S, Fei Electron Optics BV, Eindhoven, The Netherlands). All chemicals were purchased from Sigma-Aldrich. High-power micrographs collected at X8900 magnification were utilized to evaluate the number of mitochondria and the volume fraction occupied by glycogen, myofibrils, and mitochondria in cardiomyocytes. All morphometric data were blindly collected.

### 4.6. Statistical Analysis

The IBM SPSS statistical package (International Business Machines Corporation, version 24, Armonk, NY, USA) was used. Normal distribution of variables was checked by means of the Kolmogorov-Smirnov test. Data are reported as mean ± standard error of the mean (SEM). Comparisons among groups involved GLM/ANOVA for repeated measures (for cardiomyocyte contractility and Ca^2+^ transients) and non-parametric statistical test Kruskal Wallis and U-Mann Whitney test (TEM data). Differences were considered statistically significant at *p* < 0.05.

## 5. Conclusions

In conclusion, this study demonstrated for the first time a direct negative role of TMAO on cardiomyocyte contractile function and intracellular calcium handling, and suggested that Uro B-gluc can counteract these detrimental effects. The protective action of Uro B-gluc on TMAO-induced cardiac dysfunction is a new observation, which should be deeply explored as a dietary strategy in the complex TMAO scenario.

These data represent only a preliminary step, and further detailed mechanistic studies are needed in order to define the specific intracellular pathways involved in TMAO pathogenic effects and in the ability of Uro B-gluc to recover cardiac cell function. Furthermore, in vivo animal studies are required for a deeper evaluation of cardiac performance at organ level, following short and long-term diets rich in phosphatidylcholine/choline and/or carnitine as precursors of TMAO, with or without the supplementation of ETs. Finally, considering the microbial origin of urolithins after ET colonic catabolism, a potential role of these metabolites as microbial modulators or inhibitors of TMA colonic production could not be completely excluded and might be considered in future investigations. In this framework, a human intervention study should be considered as a powerful instrument to correlate the ability to produce colonic urolithins from ETs, because of the reported inter-individual variability [50], and the potential effect on TMAO circulating level.
